# Numerical investigation of respiratory drops dynamics released during vocalization

**DOI:** 10.1063/5.0059419

**Published:** 2021-08-18

**Authors:** C. Peña-Monferrer, S. Antao, R. Manson-Sawko

**Affiliations:** IBM Research Europe, The Hartree Centre, Warrington WA4 4Ad, United Kingdom

## Abstract

Release of drops from a human body has been the focus of many recent investigations because of the current COVID-19 pandemic. Indirect virus transmission from asymptomatic individuals has been proved to be one of the major infectious routes and difficult to quantify, detect, and mitigate. We show in this work a detailed and novel numerical investigation of drops released during vocalization from a thermal manikin using a large eddy simulation coupled with Lagrangian tracking of drops. The vocalization experiment was modeled using existing data from the literature for modeling exhaled airflow, emission rate, and size distribution. Particular focus was on the definition of the boundary conditions for the exhalation process. Turbulence was compared with experimental data for the near mouth region for 75 exhalation breathing cycles and showed the sensitivity of different modeling assumptions at the mouth inlet. The results provide insights of special interest for understanding drop dynamics in speech-like exhalation modes, modeling the mouth inlet boundary conditions, and providing data for verifying other more simplified models.

## I. INTRODUCTION

Spread of COVID-19 around the world^1^ has demanded urgent action from the scientific community on different fronts. One of the key aspects for controlling the pandemic is improving the understanding of the involved mechanisms for mitigating virus transmission. This is especially important for the current outbreak but also crucial for the design of spaces and ventilation systems with a focus on a post-COVID-19 era as well as for minimizing the effects of potential future pandemics. In any case, delivering effective mitigation measures^2^ for particular outbreaks or improving hygiene in indoor environments is a challenge of the highest priority.

Disease transmission through the release of droplets from the respiratory system has been traditionally divided into direct contact, indirect contact/fomite, droplet, and aerosol.^3^ Different particle size cutoffs have been used to define droplets and aerosols, as well as for defining the composition of the airborne. Traditionally, aerosols have been considered as droplet nuclei of size smaller than 5 *μ*m, and droplets bigger than 5 *μ*m as respiratory droplets. Although this provides a very general view on how droplet sizes carrying virus might be responsible for different types of virus transmission, the dynamics of this problem is subject to high variability and depends on the specific environmental factors, ventilation, and characteristics of the individuals involved.^4–6^ In the context of COVID-19, the importance of each route has been highlighted by the scientific community, e.g., respiratory drops or aerosols by Refs. 7 and 8.

The number of experimental and data-driven studies about this topic, as well as numerical simulations at different scales from laboratory to full-scale size, is wide and rapidly growing.^9^ Dynamics of drops released from nose or mouth and for different human actions and under mitigation measures have been investigated in depth in the past, both experimentally^10–21^ and numerically.^22–40^ On a larger scale, numerical simulations are also present in the literature focusing on the effect of ventilation systems on virus spread^41–55^ or the design of devices and techniques for mitigating transmission risk.^56–61^ In addition, different solutions are currently being used in commercial^62,63^ and open-source software^64^ applied to real scenarios such as airline cabins, social distancing, treatment rooms, production lines, or offices amongst others.

We focus the present study on a validation of a breathing thermal manikin with experiments and a subsequent numerical investigation of drops dynamics during the vocalization of a vowel /ɑ/. Different modeling assumptions for the exhaled flow boundary conditions at the mouth were presented and evaluated for analyzing the impact of turbulence and mean velocity profiles on the flow and drops dynamics. A comprehensive model, consisting of a high-resolution Eulerian-Lagrangian simulation with a large eddy simulation (LES) turbulence model, transport of carrier species, multi-component particle tracking, carrier/particle vapor interaction, inflow synthetic eddy boundary conditions, heat transfer, and thermal radiation was used in this work. This study was based on open-source tools and freely available data sets including computational fluid dynamics (CFD) software, CAD geometry, and data for defining the vocalization airflow boundary conditions. The paper also provides a detailed description of the model for reproducing the simulations.

The use of computational simulations in this particular work allows us to not only reproduce experimental studies from the literature, but also to extend the scope of the experiments to gain insights into physics that is difficult to capture experimentally. In the same way, this also contributes to providing cheaper experimentation in complex conditions such as virus transmission in a plane and under flight conditions.

## II. CFD MODELLING

This section describes the main aspects related to the modeling of the problem. Details about the solver, modeling of the exhalation modes, and domain set-up are provided.

### A. Model

The *reactingParcelFoam* OpenFOAM v2012 solver was used. This solver allowed us to compute in an Eulerian-Lagrangian framework, a transient, turbulent, compressible, multispecies, and multiphase particle cloud scenario. The particles are considered rigid and spherical, and rotational motion is neglected. A one-way coupling approach is used in this work given that the scenarios evaluated had a very low particle concentration. For instance, emission rates of around 41 particles per second and maximum particle sizes of 10 *μ*m were imposed. Further details about these parameters are described in Sec. II C. The solution was run with an adaptive time step based on maximum Courant numbers of 1 for the Eulerian part and 0.01 for the Lagrangian part.

#### 1. Eulerian framework

The Eulerian part consisted of a fluid phase consisting of two species, namely, air and water vapor. Turbulence was modeled using an LES approach with a Smagorinsky subgrid-scale model. The Schmidt and Lewis numbers were set to one, and turbulent transport was assumed to dominate over molecular effects leading to simplified forms of the species and enthalpy equations. The filtered governing equations for continuity [Eq. 1(a)], momentum [Eq. 1(b)], species transport [Eq. 1(c)], and energy [Eq. 1(d)] are defined below

$$\frac{\partial \rho }{\partial t}+\nabla \cdot (\rho u)=0,$$ (1a)

$$\frac{\partial }{\partial t}(\rho \;u)+\nabla \cdot (\rho \;uu^{⊺})=-\nabla p+\nabla \cdot \tau +\rho \;g,$$ (1b)

$$\frac{\partial }{\partial t}(\rho \;Y_{i})+\nabla \cdot (\rho \;uY_{i})=\nabla \mu_{\text{eff}}\cdot \nabla Y_{i}+{\dot{R}}_{i},$$ (1c)

$$\begin{array}{c} \frac{\partial }{\partial t}(\rho \;h)+\nabla \cdot (\rho \;uh)+\frac{\partial }{\partial t}(\rho \;K)+\nabla \cdot (\rho \;uK)-\frac{\partial p}{\partial t}\\ =\nabla \alpha_{\text{eff}}\nabla h+{\dot{R}}_{\text{reac}},+{\dot{S}}_{\text{rad}}, \end{array}$$ (1d)

where *ρ* is the fluid density of the mixture, *t* is time, ***u*** is the flow velocity field, *p* is the pressure, τ is the subgrid-scale stress tensor, ***g*** is the gravity vector, *Y_i_* is the mass fraction of the *i*-th species, $\mu_{\text{eff}}$ is the effective dynamic viscosity, ${\dot{R}}_{i}$ is the production rate of the given species due to reaction, *h* is the specific enthalpy, *K* is the kinetic energy, $\alpha_{\text{eff}}$ is the effective thermal diffusivity, ${\dot{R}}_{\text{reac}}$ is the heat generation by reactions, and ${\dot{S}}_{\text{rad}}$ is the energy source due to thermal radiation. Reactions are neglected, and therefore, ${\dot{R}}_{i}$ and ${\dot{R}}_{\text{reac}}$ are both zero for this work. The radiative heat transfer in the system for calculating ${\dot{S}}_{\text{rad}}$ is modeled using the P1 model.^65^ Finally, a one-way coupling is used, and no source term representing the particles effect in the flow is considered in the equations above.

We use OpenFOAM implementation of the Smagorinsky model to close subgrid-scale stress tensor in the equations. This is given by the following equations:

$$\tau -\frac{\text{tr}(\tau )}{3}I=\rho (\nu_{t}+\nu )D,$$ (2)

$$\nu_{t}=C\Delta \sqrt{k}$$ (3)

with *ν* being kinematic viscosity. *k* is given by the solution of the quadratic equation $ak^{2}+bk+c=0$, where $a=\frac{C_{e}}{\Delta },\;b=\frac{2}{3}\text{tr}(D),c=2C_{k}\Delta (\text{dev}(D):D)$ and $D=\frac{1}{2}\left(\nabla u+u^{T}\right)$. For the meaning and values of each coefficient, we point the reader to OpenFOAM documentation pages.^66^ We also select the filter length Δ as equal to the volume-based length-scale.

Mixture properties are calculated from the species mass fraction and the temperature-dependent material properties of air and water vapor components provided by a thermochemical database.^67,68^ Air is composed of ingredients with the following weights: 78.084% N_2_, 20.9476% O_2_, 0.9365% Ar, and 0.0319% CO_2_.

#### 2. Lagrangian framework

The Lagrangian part was formed by multiphase drop particles composed of liquid water and a non-evaporable solute phase composed of NaCl. The particles were seeded at the patch representing the mouth opening, with a defined particle rate and size distribution. The motion of the *j*-th drop was computed by integrating Newton's second law of motion,

$$m_{j}\frac{du_{j}}{dt}=f_{j}^{g}+f_{j}^{d}+f_{j}^{l},$$ (4)

where *m_j_* stands for the *j*-th drop mass, *u_j_* is the instantaneous drop velocity, $f_{j}^{g}$ is the gravity force, $f_{j}^{d}$ is the drag force, and $f_{j}^{l}$ is the lift force. *m_j_* is the total mass of the drops composed of evaporable ($m_{e}$) and non-evaporable ($m_{\text{ne}}$) components. Carrier phase values at the center of the particles during the coupled solution were passed as cell point interpolated values for all the variables including *Y_i_* and are used for calculating the particle forces and the vapor interaction model.

Small spherical drops are expected for the conditions used in this work, and consequently, the drag force is modeled using the Schiller–Naumann^69^ drag coefficient correlation. The lift force due to local shear flow is modeled using the Saffman–Mei model first derived by Saffman^70^ and later generalized by Mei.^71^

The interaction of the drops with the carrier vapor phase was modeled as described by Chen *et al.*^72^ The mass change of the evaporable component, $m_{e}$, is defined as

$$\frac{dm_{e}}{dt}\approx -n_{e_{j}}A_{j},$$ (5)

where $n_{e_{j}}$ and *A_j_* are the average mass flux of the evaporable component and the surface area of the *j*-th drop, respectively. $n_{e_{j}}$ is modeled using the Fuchs–Knudsen correction applied for water in the dilute NaCl solution described by Chen *et al.*^72^

Particle temperature is calculated using the following equation:

$$m_{j}C_{p,j}\frac{dT_{j}}{dt}=h_{j}A_{j}(T_{j}-T_{c})-n_{e_{j}}L_{e_{j}}A_{j},$$ (6)

where $C_{p,j}$ is the specific heat and *h_j_* is the convection heat transfer coefficient, and *L_e_* is the evaporatable latent heat of the *j*-th drop. *h_j_* is calculated using the Ranz–Marshall correlation^73^ for the Nusselt number. *T_j_* and *T_c_* are temperatures of the *j*-th drop and carrier phase, respectively.

The material properties of the multiphase droplet are defined as liquid water with data from the thermophysical database,^67,68^ and NaCl with values of molar mass 58.4 kg kmol^−1^ and density of 2165 kg m^−3^.

### B. Domain, mesh, and boundary conditions

The experiments performed by Jiang *et al.*^74^ and Feng *et al.*^75^ were used to set up our case, evaluate different inlet conditions, and validate the fluid dynamics of the exhalation for different phases of the sinusoidal wave. In the experiment, a manikin mockup was used to measure mean velocity and turbulence fields for exhalation with a two-dimensional time-resolved particle image velocimetry. The manikin was a thermal body comprising head, trunk, and legs. A circular opening of diameter 12 mm was used as a mouth for the experiment. The mockup was placed in an enclosed chamber with an indoor air temperature maintained at 20 ± 1 °C by a packaged air conditioner. Two conditions were evaluated in the experiment, an isothermal condition with manikin and exhaled air not heated, and a heated condition with manikin and exhaled air heated to 34 °C. The temperature was measured inside the experimental mockup, at 10 cm from the manikin, reporting average values of around 20.83 °C and 24.39 °C for the isothermal and heated conditions, respectively.

The experiment was focused on the investigation of the exhaled flow but without considering drops. The approach followed in our work is using this configuration and measurements for calibrating our computational model and then performing virtual experiments of drops released from the vocalization of vowel /ɑ/. The scope of the current work is evaluating the vocalization virtual experiment for the same experimental set-up. Future investigations will cover this process in other domains such as in forced ventilated scenarios.

In our preliminary studies, we discovered that the flow was highly sensitive to the specification of inlet boundary conditions. We formalize three approaches to evaluate their influence on the exhaled jet and turbulent characteristics, namely: (1) uniform profile, (2) radially varying profile, and (3) radially varying profile with random perturbation. For radial variation, an empirical profile is used with values taken from experimental data. Laminar and turbulent inlet conditions at the inlet were imposed and labeled as LAM and TURB, respectively. At the same time, inlet conditions with uniform mean values or radial profile for velocity at the mouth were tested and labeled as MEAN and PROF, respectively. This allowed us to investigate the influence of velocity definition on jet development and drops dynamics. For instance, we can quantify how local and temporal velocity plays a role in the evolution of the jet for determining the momentum-dominant and buoyancy-dominant jet phases as well as capturing jet instabilities as the exhalation process evolves.

**FIG. 1.**
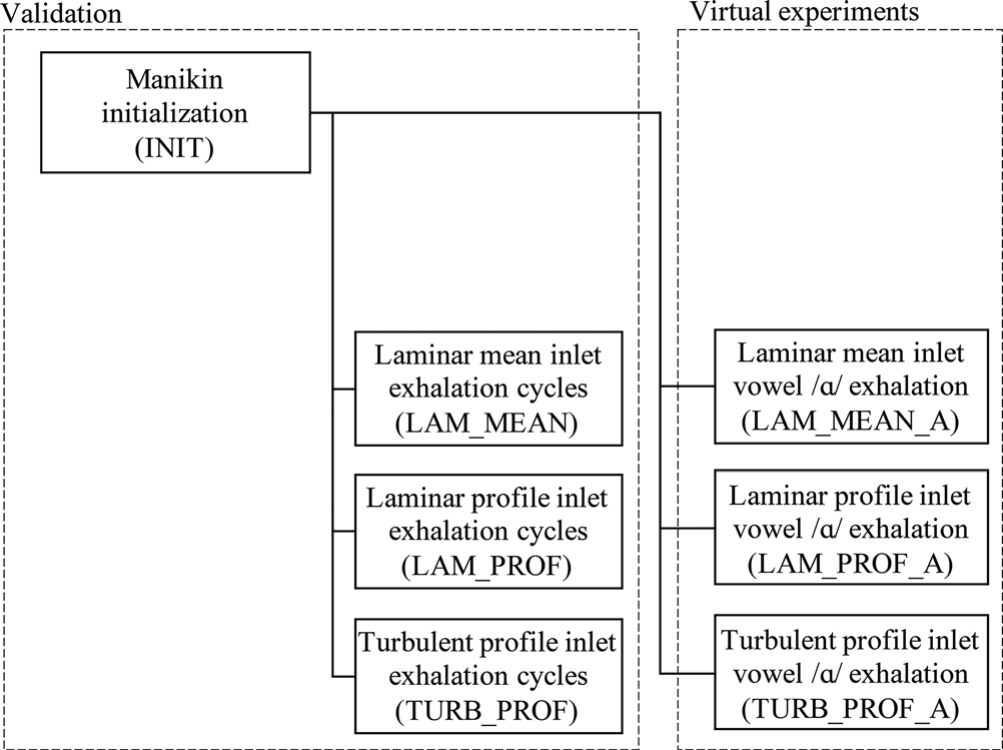
Overview and labels of the simulations.

Figure 1 shows an overview of the approach. A first simulation, INIT, was run to initialize the field before any exhalation process took place. Three simulations, LAM_MEAN, LAM_PROF, and TURB_PROF, were performed and compared with the experimental data of different exhalation breathing cycles. Finally, the vocalization virtual experiments were run using the same three inlet approaches with the labels LAM_MEAN_A, LAM_PROF_A, and TURB_PROF_A.

**FIG. 2.**
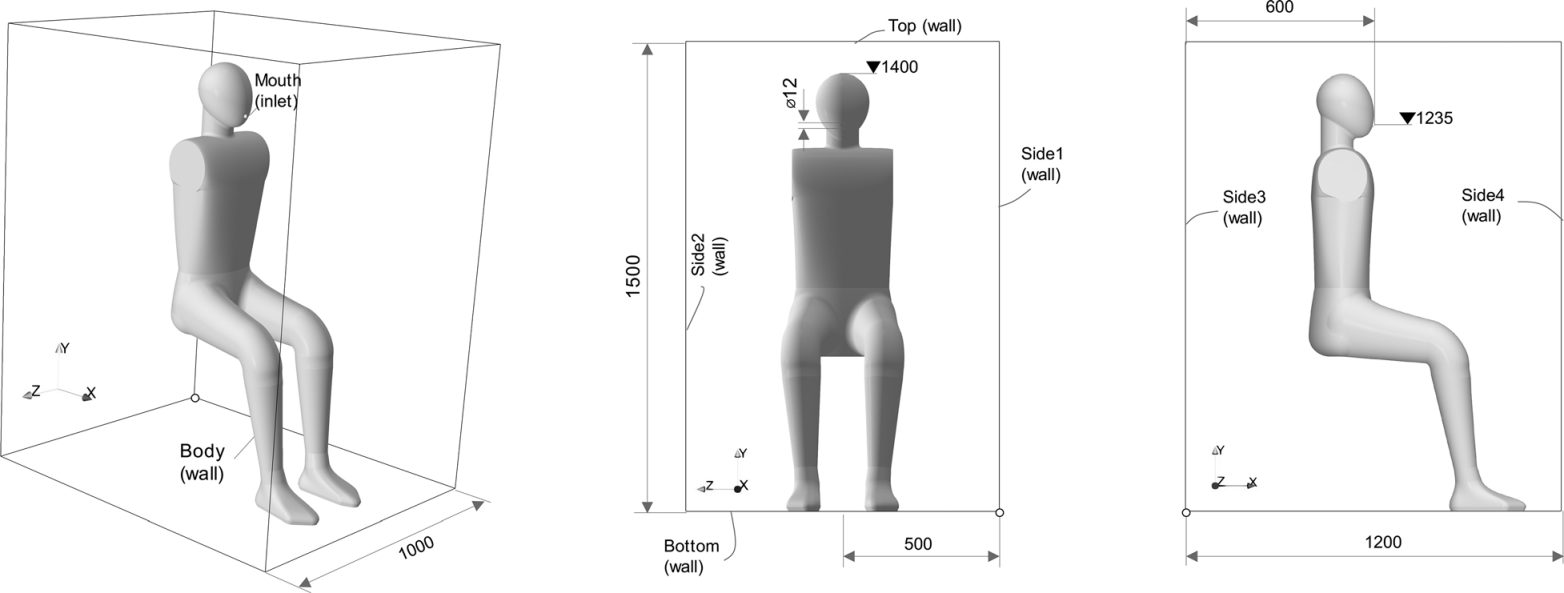
Schematic representation of the domain including dimensions and patch label (units in millimeters).

The computational domain consisted of a manikin in a rectangular box of size 1 × 1.5 × 1.2 m^3^ and with all side patches defined as walls to match the experimental configuration. Figure 2 depicts this configuration schematically.

The manikin body was represented by a freely available 3D CAD^76^ model scaled to match the 1.4 m sitting height of the manikin in the experiment. A circular opening of 12 mm of diameter was made on the geometry for creating the mouth patch. We removed the arms from the original CAD to match the one from the experiment. Our geometry resulted in a surface area of 1.36 m^2^, which is only 1.6% higher than the experimental one with 1.339 m^2^. A total sensible heat loss of 75 W was reported in the experiment and reported in segments with heat losses of 7.4 W for the head, 25.6 W for the trunk, and a total of 42.0 W for the legs. These values were imposed as heat fluxes on the CFD model for each segment to match the sensible heat losses. Temperature at the side and top walls was fixed to 20.83 °C, corresponding to the measurements inside the mockup for the isothermal condition. This value is also in the 20 ± 1 °C range reported for the chamber outside the mockup, for both conditions. The temperature at the bottom wall was defined as adiabatic.

Initial mass fractions in the domain for water vapor, $Y_{d,H_{2}O}$, and air, (1-$Y_{d,H_{2}O}$), are defined in the simulation based on relative humidity. A relative humidity (RH) of 30% for an initial temperature of 24.39 °C, corresponding with the average value measured inside the mockup, was used to define the initial values of mass fractions in the domain. Details about temperature and humidity for exhalation are described in Sec. II C.

**FIG. 3.**
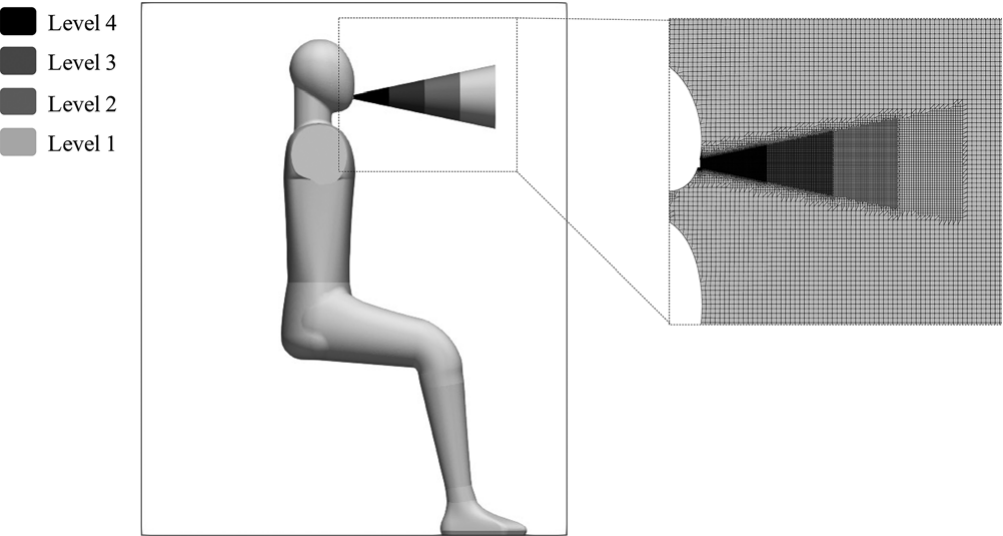
Mesh refinement levels and near mouth mesh zoom.

The mesh was generated using *blockMesh* and *snappyHexMesh* OpenFOAM tools, generating a 7.2 × 10^6^ cubic cell element with different levels of refinement imposed on the jet region. This resulted in a cell size of 7 mm for the background mesh and 0.4375 mm for level 4 corresponding with the region near the mouth. Figure 3 provides details about the refinement levels and mesh for the near mouth region.

The cell size near the mouth is smaller than the one used in similar works on LES breathing simulations. For instance, Khosronejad^34^ used a resolution of 0.5 mm near the mouth and verified that their simulations resolved almost all relevant scales of motion representing a high-fidelity simulation of human breathing. This level of resolution also provided in our work a good agreement compared with the experiments.

### C. Exhalation modeling

In this section, we describe the conditions for the exhalation modes used in this work, namely, exhalation cycles and vocalization. The former is based on a set of consecutive exhalation cycles used for comparing simulation results with experimental data. The vocalization exhalation mode is represented with data for the pronunciation of the vowel /ɑ/ by a female. Sections II C 1–II C 4 describe the boundary conditions employed. In particular, variables such as velocity, turbulence, temperature, species, drop emission rate, and particle size at the mouth patch are described.

#### 1. Inlet mean velocity

The slight complication in the context of exhalation is that both the underlying (constant or radially varying) profiles and the perturbation are function of time. The flow rate at the mouth, $Q_{m}$, is given as follows:

$$Q_{m}(t)=k(t)U_{m,c}(t),$$ (7)

where $U_{m,c}$ is the streamwise inlet velocity at the centerline. *k* is an auxiliary variable representing the ratio between the centerline velocity and flow rate, i.e.,

$$k(t)=2\pi \int_{0}^{r_{m}}\frac{U_{m}(r,t)}{U_{m,c}(t)}rdr,$$ (8)

where $U_{m}$ is the streamwise velocity at a given radial coordinate, *r*. The radius of the circular opening, $r_{m}$, is fixed to 6 mm.

**FIG. 4.**
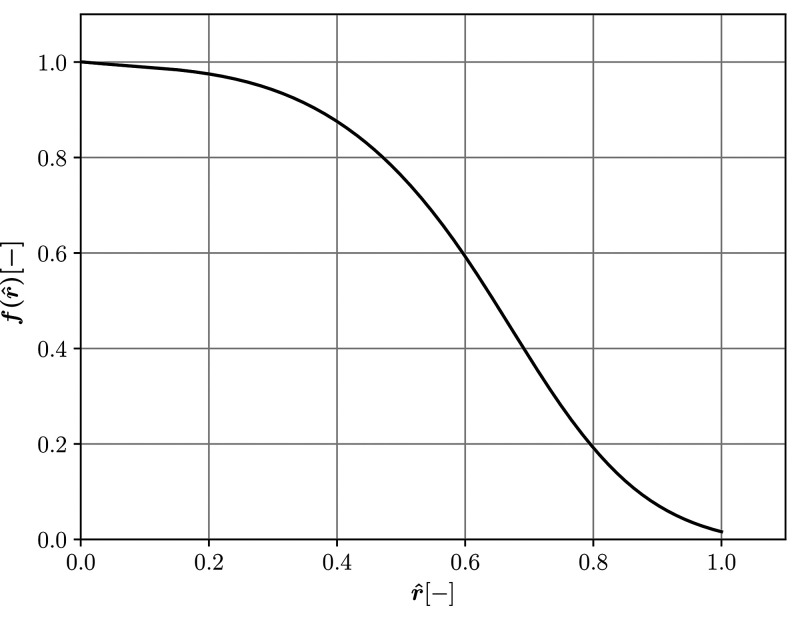
Radial distribution of $f(\hat{r})$ as a function of the normalized mouth radius. This radially varying empirical profile is used in LAM_PROF, LAM_PROF_A, TURB_PROF, and LAM_PROF_A.

The ratio $U_{m}(r,t)/U_{m,c}(t)$ was found to be constant in time in the experiments of Feng *et al.*^75^ for all the phases of the sinusoidal wave for a 6 mm radius circular opening. This allows us to define the mean velocity at any radial position as a function of its value at the centerline,

$$U_{m}(r,t)=U_{m,c}(t)f(\hat{r}),$$ (9)

where $\hat{r}$ is the normalized radius, $\hat{r}=r/r_{m}$ and $f(\hat{r})$ is passed to the code as a lookup table with linear interpolation using experimental values from Feng *et al..*^75^ The latter is presented in Fig. 4 and applied to LAM_PROF, LAM_PROF_A, TURB_PROF, and LAM_PROF_A.

In the case of a radially constant inlet function, $f(\hat{r})$ in Eq. (9) needs to be replaced with a single value and the resulting velocity is no longer position dependent leading to an expression

$${\bar{U}}_{m}(t)=\frac{\int_{A}U_{m}(r,t)dA}{A}=2U_{m,c}(t)\int_{0}^{1}f(\hat{r})\hat{r}d\hat{r}.$$ (10)

Velocity defined in this way is used in scenarios labeled as LAM_MEAN and LAM_MEAN_A.

The centerline velocity $U_{m,c}$ is defined as a rectified sinusoidal wave for modeling the exhalation cycles. A least squares fit to experimental data from Feng *et al.*^75^ results in the following functional form:

$$U_{m,c}(t)=\left|4.15\;\text{sin}\;(1.527t)\right|,$$ (11)

which gave the sinusoidal period of 2.0573 s.

Modeling the airflow exhaled during speaking is a very challenging task that requires accounting for different phenomena occurring upstream from the mouth such as the interaction of various components of the vocal tract, as well as the dynamics of the mouth opening. In addition, the variation between different subjects or activities is very pronounced. For instance, an average airflow for normal speech can be around 0.15 l/s for a passage reading,^13^ but can reach significantly higher peak values for a soprano singing up to 2 l/s.^77^

We focus in this work on reproducing the pronunciation of the vowel /ɑ/ at the mouth boundary condition in the experimental set-up investigated. This allows us to retrieve enough information from the literature to define the glottal flow over time, particle rate, and drop particle distribution for this condition.

The open-source repository OPENGLOT^78^ provides a collection of data used for the evaluation of glottal inverse filtering algorithms. These data contain synthetic and recordings of natural vowel production and provide speech pressure signal, glottal flow, and glottal area data for different phonation and fundamental frequencies, *F*_0_. This allows us to combine data for the glottal flow with particle rates and size distributions from Ref. 79 for a representative female vocalizing vowel /ɑ/.

The data in OPENGLOT provide normalized values of the glottal flow waveform and need to be scaled or calibrated with the actual average flow. We use data from Repository III as it provides data for different fundamental frequencies for a female pronouncing the vowel /ɑ/. Repository III provides glottal excitation and speech pressure signals generated from a 3D printed vocal tract replica by Liljencrants–Fant (LF) excitation via a loudspeaker. In particular, data from this repository at *F*_0_ 250 Hz were used in this work. The variability on the airflow curve and drop emission rate for different human actions or gender, speech or loudness amongst others, indicates that an uncertainty quantification would be needed to reproduce human actions better. However, the scope of this work is to provide representative values of vocalization to perform the aforementioned virtual experiments to understand drops dynamics for this exhalation mode. Further investigations will be focused on extending this study to a wider range of conditions.

A review of the literature about measurements of airflow using a Rothenberg mask^77^ allowed us to set up the right parameters of calibration. Gupta *et al.*^13^ measured flow rate values when articulating F, E, C, O, S, and T letters of the alphabet. This work provides values of average flow rate as a function of body surface area for males and females. However, the differences in the airflow rate for the different letters could be significant. For instance, F, S, and T were found to be the letters that provided the maximum exhalation flow rates. This is in fact in accordance with the results showed by Rothenberg *et al.*^77^ Holmberg *et al.*^80^ provided values for characterizing the glottal flow such as average flow, peak flow, and minimum flow among others for the airflow of vowel /æ/. Values of average flow of 140 ml/s, peak flow of 230 ml/s, and minimum flow of 80 ml/s were reported for normal pitch in females with a mean *F*_0_ of 205 Hz and standard deviation of 24 Hz. Similarly, Orlikoff^81^ reported values of 133.9 ± 9.4 ml/s for a sustained phonation of vowel /ɑ/ at *F*_0_ 212.7 Hz for females. On the other hand, Alku^82^ indicated values of around 100 ml/s for the mean minimum flow in speech produced using normal loudness for both genders, with values for males slightly higher. Using these data, we scaled the glottal flow waveform from OPENGLOT to match a threshold of minimum flow of 100 ml/s and an average flow of 140 ml/s resulting in a peak flow of 240 ml/s.

**FIG. 5.**
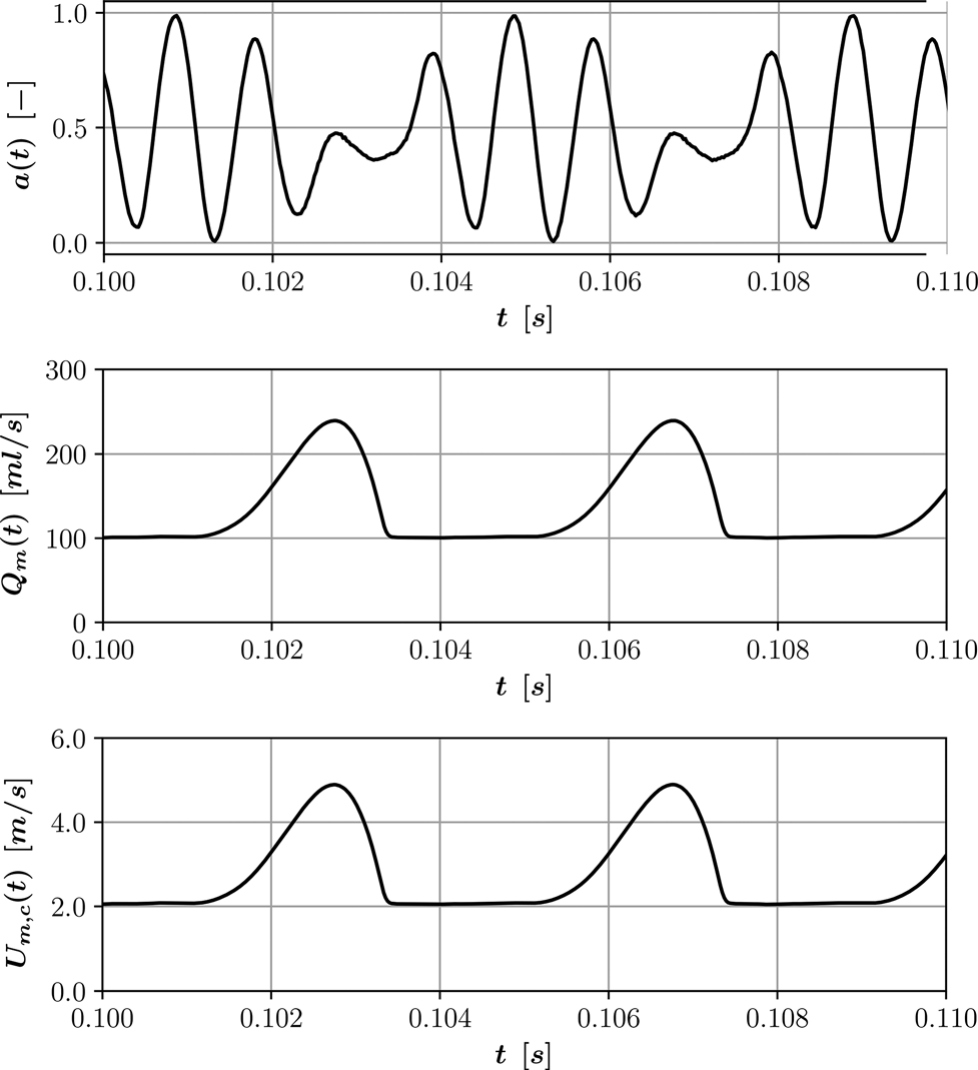
Normalized pressure signal (arb. units), glottal flow rate, and centerline velocity for the vocalization of the vowel /ɑ/ virtual experiment.

The data provide intervals of 0.2 s that we concatenated to reach a total time of 10 s. The flow rate was used to calculate the temporal evolution of $U_{m,c}$ using the flow rate definition in Eq. (7). Figure 5 shows values for the normalized pressure signal, glottal flow, and calculated values of the centerline velocity for a window of 0.01 s.

#### 2. Turbulence

Turbulent fluctuations during exhalation have been observed experimentally for different exhalation modes using schlieren and shadowgraph imaging experiments,^7,83^ simulations,^84^ or a combination of both.^85^ To represent them in an LES, we propose an effective method for generating correlated, turbulence-like perturbations. We compare the results with a purely laminar inlet, as shown in Fig. 2.

The simplest decomposition of turbulence is into a mean and a fluctuating component. The fluctuation can be simulated by injecting white noise, but this does not guarantee spatial and temporal correlations required for sustained turbulence, leading to quick relaminarisation.^86,87^ To solve this issue, Jarrin *et al.*^88^ developed a synthetic eddy method (SEM), which defines the fluctuating field as a combined effect of randomly generated vortices, whose properties depend on the target turbulence statistics. To inject turbulent-like motion, we employ the divergence-free synthetic eddy method (DFSEM) of Poletto *et al.*,^89^ which enforces mass continuity, but sacrifices the ability to reproduce all realizable Reynolds stresses. This approach follows the same overall procedure followed by Jarrin *et al.*^88^ for defining and convecting the synthetic eddies that represent the velocity fluctuations but provides a divergence free fluctuating velocity field. This can be written in Cartesian tensor notation for any point of the plane as follows:

$$u_{i}^{\prime }(x)=\sqrt{\frac{1}{N}}\sum_{k=1}^{N}\sigma_{i}^{k}[1-{\left(d^{k}\right)}^{2}]\varepsilon_{\mathrm{ijl}}r_{j}^{k}\alpha_{l}^{k},$$ (12)

where *N* is the number of eddies, $\sigma_{i}^{k}$ is the length scale for the *k*-th eddy in the *i* direction, $d_{k}=\sqrt{{\left(r_{j}^{k}\right)}^{2}},\;\varepsilon_{\mathrm{ijl}}$ is the Levi-Civita symbol and to simplify the expression the summation over *j* and *l* indices is implied. Furthermore, $r_{j}^{k}=\frac{x_{i}-x_{i}^{k}}{\sigma_{i}^{k}},\;x_{i}^{k}$ is the location of the center of the eddy, and $\alpha_{l}^{k}$ are random numbers with zero averages representing the eddy intensity. The eddy intensity is calculated to return the imposed Reynolds stress statistics for a defined series of length-scale ratios. The reader is referred to Poletto *et al.*^89^ and Poletto *et al.*^90^ for further details about the implementation.

The inlet specification for our case requires knowledge of spatiotemporal distribution of the Reynolds stress tensor, $R_{m}(r,t)$. Different experimental^91,92^ and direct numerical simulations (DNS) studies^93–96^ cover situations when statistics changeover space and time, but the type and magnitude of change do not match the characteristics of the exhalation process. Moreover, exhalation depends on the upstream respiratory tract which, to the best of the authors' knowledge, has not yet been studied in sufficient detail, e.g., $R_{m}(r,t)$ have not been reported.

In the absence of relevant data, we assume the off diagonal stress components to be negligible but recommend evaluating nonzero values in any future study. This leads to

$$R_{m}(r,t)={\left(I_{m}U_{m,c}(t)\right)}^{2}I,$$ (13)

where **I** is the identity tensor and $I_{m}=u_{\text{rms}}^{\prime }/U_{m,c}$ is the turbulence intensity with mean centerline velocity as a reference. $I_{m}$ was fixed to 0.16 as a first approximation based on measurements reported by Feng *et al.*^75^ These results demonstrate that the flow was turbulent for the whole exhalation process. The authors reported contours of spatial variation of $I_{m}$ near the injection regions for the early, intermediate, and late phases of the sinusoidal wave. The contours show a near homogeneous radial distribution at the mouth and especially for the late phase at φ = 18. The intermediate phase, φ = 10, had slightly lower values at the core, with peak values similar in magnitude to the values at phase 18. Finally, the early phase, φ = 2, had lower values than the other two phases. As the influence of turbulence on jet development appeared to be rather marginal in the early phases, we chose a constant value of 0.16, which is representative of the intermediate and late phases. This resulted in overall good results for the exhalation cycle as revealed by comparison with all three phases.

In line with the modeling assumption expressed in Eq. (13), we limit ourselves to spherical eddies with magnitudes varying over time. This is achieved by fixing the ratio of all eddy length scales to one in the principal stress coordinate system. The turbulent length-scale was set to three times the mesh scale for producing flow structures that persist through the domain.^97^ Moghadam *et al.*^98^ investigated the effect of this parameter in a channel flow, finding a good agreement with 3 cells per eddy for capturing non-decaying turbulence fluctuations. Increasing this value to 5 required more computational effort but did not demonstrate a significant improvement in the flow characteristics. Therefore, this set-up provides a good approximation of the injection flow behavior, which is corroborated by the experimental comparison presented in Sec. III B.

Although a spatiotemporal definition of $I_{m}$ would be ideal, the reference did not provide enough data for this. A constant value allowed us to perform this study and compare the results with laminar conditions but also provided very good agreement as shown in Sec. III A. Further numerical and experimental investigations are recommended in this regard to improve the definition of the turbulent characteristics and evaluate its impact on results accuracy.

#### 3. Particle rate, size distribution, and drop mass fractions

Drops were seeded spatially randomly distributed on the mouth patch for the vocalization virtual experiment with a given size distribution, particle rate, and mass fractions of the composition. The initial velocity of the particles was set to match the carrier phase velocity at the given seed point over time.

The investigation performed by Asadi *et al.*^79^ showed a linear correlation of the particle emission rate during speech with the amplitude of vocalization. On the other hand, particle size distribution was shown independent of vocalization loudness. An important variability between participants was observed in that work, showing that a small fraction of individuals behaved as superemitters and releasing an order of magnitude more particles.

Despite the variability between different individuals, we focus our investigation on the data of a representative female participant (F4 in Asadi *et al.*^79^) for whom more data were reported for vocalization of /ɑ/. In particular, data about particle emission rate as well as particle size distribution for that participant were shown. This reference shows the following correlation between particle rate, ${\dot{n}}_{m}$, and root mean square amplitudes of the sound pressure signal, $a_{m}$ (in arbitrary units) for participant F4 and vowel /ɑ/,

$${\dot{n}}_{m}=151.12a_{m}-1.9062.$$ (14)

Also, the same work reported a linear correlation for $a_{m}$ and the fundamental frequency, *F*_0_ (Hz), for the same representative participant:

$$a_{m}=(F_{0}-188.69)/217.57.$$ (15)

In Sec. II C, we defined the airflow for a specific fundamental frequency *F*_0_ 250 Hz. Using Eqs. (14) and (15) values of $a_{m}$ 0.282 and ${\dot{n}}_{m}$ 41 particles per second are obtained.

**FIG. 6.**
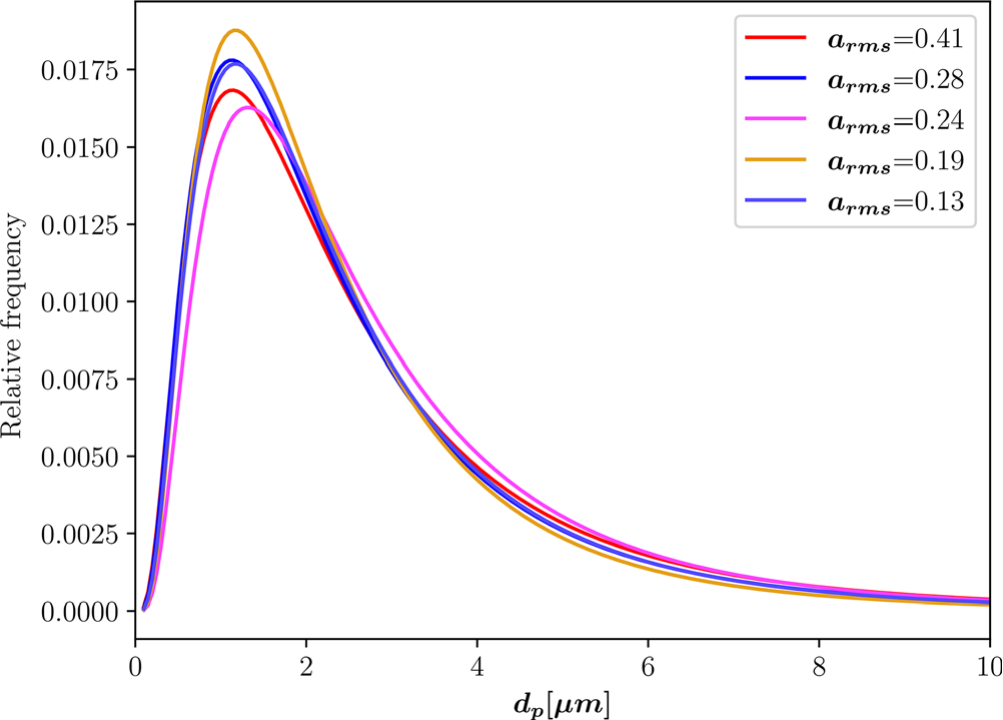
Particle size distributions for different amplitudes for a vowel /ɑ/ vocalization of a female subject.

Regarding the particle size distribution, we show in Fig. 6 the measured sizes for different amplitudes for F4 as relative frequencies. This shows how the size distributions were similar for different vocalization loudness. We use the curve for $a_{m}$ 0.28 from this figure as it is the closest one to our value of $a_{m}$ 0.282. This is a log-normal distribution with mean 0.6951 *μ*m and standard deviation 0.7577 *μ*m.

It is important to note that the size particles for this case are fixed to be smaller than 10 *μ*m. Considering a threshold of 5 *μ*m for the settling size, some of these particles would, in principle, deposit, and others would become part of the so-called aerosol. However, as a result of the evaporation process, all the particles reached their equilibrium size before 0.15 s after being seeded resulting in a maximum size of around 5 *μ*m. Note that none of the particles settled under the conditions of this experiment.

Finally, the mass fractions of water and NaCl on the drops were calculated from the volume of the sizes generated randomly from the log-normal probability density function. Sputum droplets consist of 1%–10% of their volume of solid solutes.^99,100^ Oliveira *et al.*^32^ considered the presence of different concentrations of NaCl, proteins, and surfactants in the liquid to theoretically estimate its influence on evaporation and viral transmission. We consider in our work a dilute NaCl solution with 5% NaCl in volume for each drop without accounting for the presence of different protein concentrations or surfactants. Our work provides insights into the breath jet interacting with the drops with a high-resolution LES. The effect that different combinations of protein and surfactants is beyond the scope of this paper.

#### 4. Temperature and humidity

Humidity is a key parameter for reproducing accurate drop evaporation rates as well as for determining the fluid dynamics of the plume. The experiments were performed with an exhalation temperature at the mouth, *T_m_*, of 34 °C but the humidity was not reported. Also, air injected through the mouth was not humidified to represent human mouth conditions. Mass fractions of water vapor at the mouth, $Y_{m,H2O}$, for LAM_MEAN, LAM_PROF, and TURB_PROF were defined to match the domain values $Y_{d,H2O}$. For LAM_MEAN_A, LAM_PROF_A, and TURB_PROF_A, we imposed a humid boundary condition with 99% of relative humidity to reproduce a more realistic exhalation process.

## III. RESULTS

This section describes the results for the scenarios and conditions described previously. An overview of the simulations and parameters used for defining the boundary conditions is shown in Table I.

**TABLE I:** Overview of boundary and initial values.

	*T_d_*	$Y_{d,H_{2}O}$ (RH)	*T_m_*	$Y_{m,H_{2}O}$ (RH)	*U_m_*	*I_m_*	*n_m_*
Label	(°C)	(% [%])	(°C)	(% [%])	(m/s)	(−)	(1/s)
INIT	24.39	0.572 (30)	⋯	⋯	⋯	⋯	⋯
LAM_MEAN	⋯	⋯	34	0.572 (17)	Eqs. (10) and (11)	⋯	⋯
LAM_PROF	⋯	⋯	34	0.572 (17)	Eqs. (9) and (11)	⋯	
TURB_PROF	⋯	⋯	34	0.572 (17)	Eqs. (9) and (11)	0.16	⋯
LAM_MEAN_A	⋯	⋯	34	3.344 (99)	Eq. (10), Fig. 5	⋯	41
LAM_PROF_A	⋯	⋯	34	3.344 (99)	Eq. (9), Fig. 5	⋯	41
TURB_PROF_A	⋯	⋯	34	3.344 (99)	Eq. (9), Fig. 5	0.16	41

The simulations were run on a high-performance computer system built on top of an IBM POWER9^101^ processor and with 512GB RAM/node, 2 × 3.86 GHz CPU, 44 cores/CPU, and NVIDIA Tesla V100 SXM2 16GB graphical processing units (GPUs) with RHEL 7.6. An in-house GPU accelerated version was used for these simulations. The sparse approximate inverse preconditioner^102^ for the pressure equation was taken from RapidCFD implementation,^103^ and the scalar-field solver was written in CUDA. We leveraged the neighboring of ranks to expose asynchronous execution opportunities to reduce latencies at scale using the NVIDIA Collective Communication Library (NCCL). Note that this implementation performs better, but the calculation is the same as found in standard OpenFOAM. An overview of the physical simulation time and computational wall clock time for each of the simulations is shown in Table II. The difference in wall time between cases, excluding INIT, was due to the adaptive time step strategy used and based on the Courant number. This produced bigger time steps for simulations with smaller velocity values at the inlet, such as the ones imposed for simulations with mean velocity at the inlet (LAM_MEAN and LAM_MEAN_A). The simulations with a given radial profile had higher values at the core of the patch with the turbulent one producing the highest instantaneous peak values of the three.

**TABLE II.** Overview of physical simulation time and computational wall time.

Label	Physical time (s)	Wall time (h)
INIT	200.0	60.25
LAM_MEAN	154.3	78.10
LAM_PROF	154.3	124.54
TURB_PROF	154.3	143.85
LAM_MEAN_A	10.0	11.60
LAM_PROF_A	10.0	16.37
TURB_PROF_A	10.0	16.72

### A. Domain initialization

The INIT simulation was run for 200 s until the average temperature of the domain and instantaneous values at a monitoring point of temperature and velocity were statistically stable. The monitoring point was placed at 10 cm from the chest of the manikin to match the experimental set-up. In the experiment, a temperature sensor was used with a data logger to record data over time. These data showed that the temperature was constant during the experiment with a fluctuation less than 0.3 °C.

**FIG. 7.**
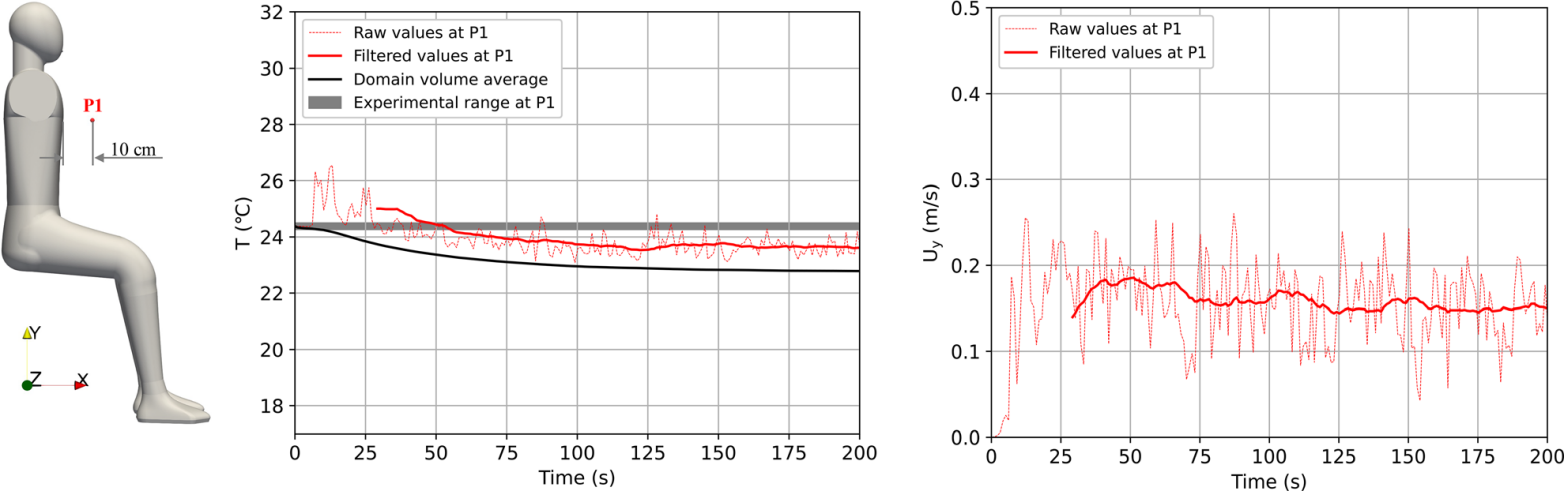
Temperature and velocity monitoring points.

We show in Fig. 7 the simulated temperature and velocity evolution over time as well as the experimental range of the temperature in the experiment. The figure depicts the volume average of the whole domain as well as raw and filtered values at the monitoring point P1. The raw values were obtained at a period of 0.01 s, while the filtered data consisted of a moving average with a 30 s window. The results showed that velocity and temperature were constant over time after around 125 s. The mean temperature was slightly below the experimental range. There are two main factors that might have influenced this result: (a) a discrepancy between the humidity imposed in the simulation and the one in the experiments producing different heat transfer rates and (b) a discrepancy between the manikin shape close to the monitoring point in the simulation and experiment producing this difference in temperature at this point. The fluctuations in the simulations were higher than the ones reported in the experiments. This might also be caused by the difference in the acquisition of the data between experiments and simulations. However, details such as acquisition frequency or filtering techniques were not reported in the reference. Ultimately, our results showed that moving average values were reasonably close to the experimental values and that they reached an equilibrium. Therefore, this simulation can be used to start the mouth breathing exhalation cycles and vocalization scenarios.

### B. Fluid dynamics validation and sensitivity of inlet boundary conditions

**FIG. 8.**
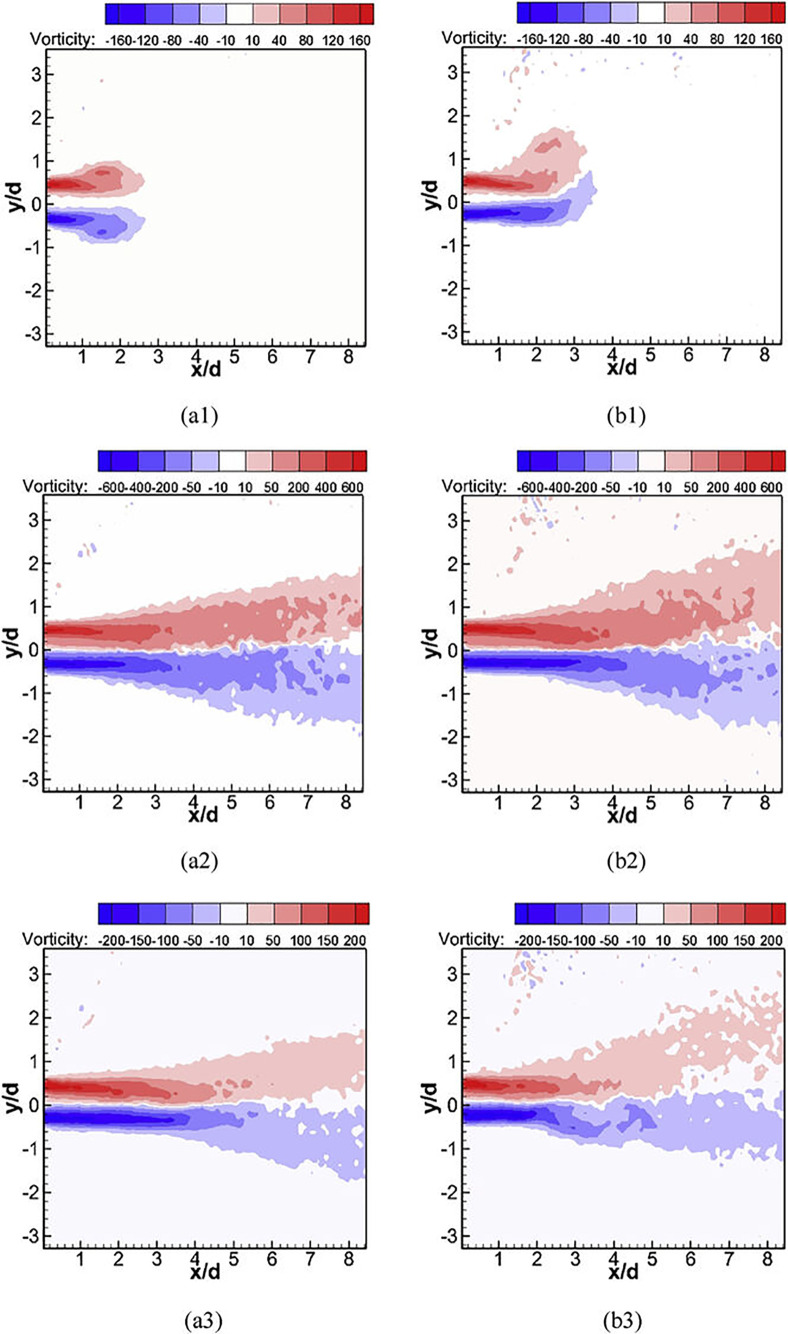
Experimental contours of phase-averaged vorticity under two conditions: (a1-3) Isothermal condition; (b1-3) Heated condition; (a1/b1) phase 2; (a2/b2) phase 10; (a3/b3) phase 18. Reprinted with permission from Feng *et al.*, Build. Environ. **94**, 683. Copyright 2015 Elsevier.

This section shows the results of the simulations used to validate the turbulent dynamics of the exhaled flow. The calculation of phase-averaged vorticity was performed during the 75 exhalation cycles of the experiment. This allows the average values to be computed consistently with their in-phase timing, accounting for instantaneous interaction between breath and body plumes. Figure 8 shows experimental phase-averaged vorticity values for the different phases measured and reproduced from Feng *et al.*^75^

The figure includes values for both isothermal (a1, a2, and a3) and heating (b1, b2, and b3) experiments. The influence of the thermal effects on the jet for different phases can be easily appreciated from these images, producing an upward shift on the vorticity field.

**FIG. 9.**
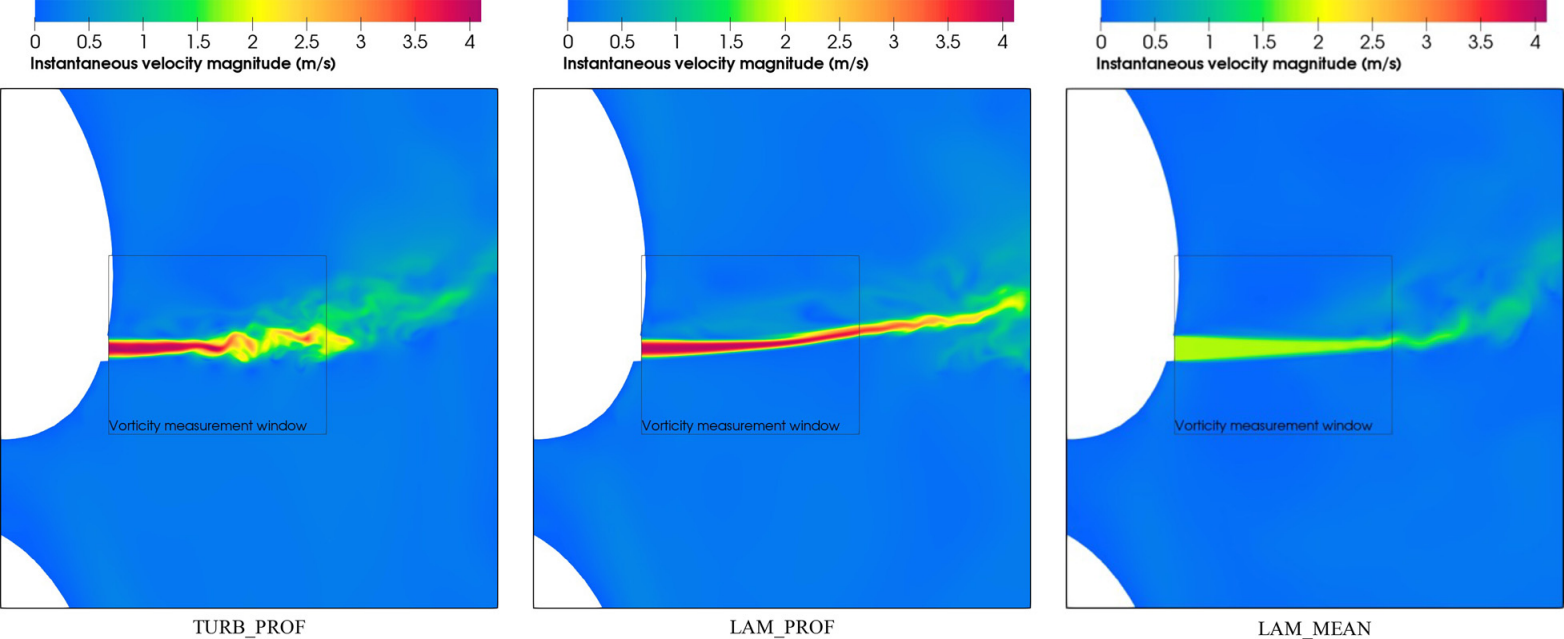
Instantaneous velocity contours for LAM_MEAN, LAM_PROF, and TURB_PROF at phase 10 and cycle 75.

In Fig. 9, we show results of the computations for instantaneous velocity contours for the intermediate phase (phase 10) at cycle 75 for LAM_MEAN, LAM_PROF and TURB_PROF. This image shows how the maximum velocity is lower for LAM_MEAN, which results from the radially averaged values imposed at the inlet. In contrast, for LAM_PROF and TURB_PROF, the maximum velocity is encountered at the core of the jet. As a result, the breath plume developed further for these latter two cases. Moreover, TURB_PROF develops early oscillatory motion in comparison with LAM_PROF, demonstrating the influence of the turbulent fluctuations. Figure 9 presents a visual comparison of the three approaches evaluated and gives an initial idea on how they might impact a scenario with drops injection.

**FIG. 10.**
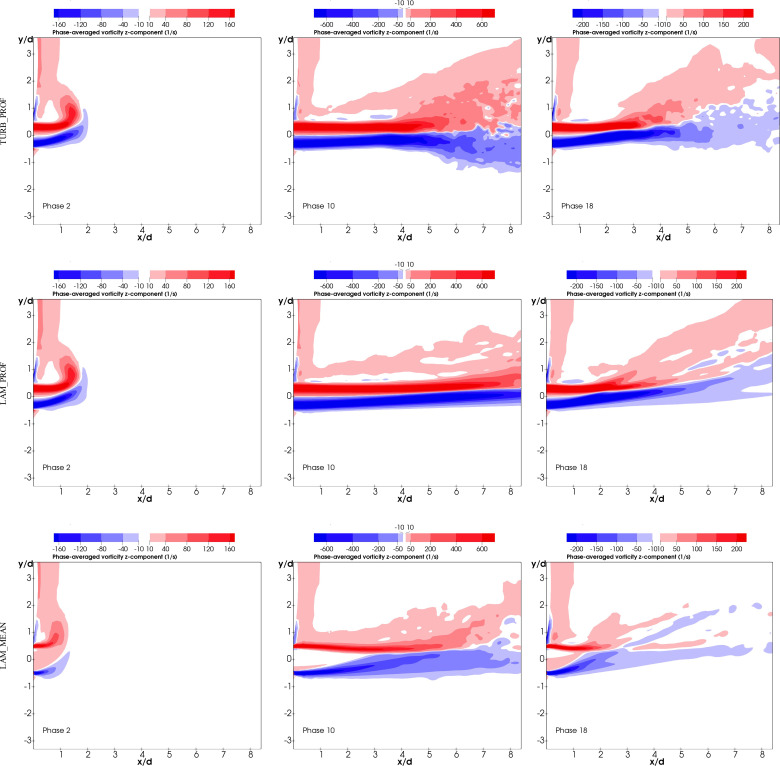
Computational contours of phase-averaged vorticity for phase 2, phase 10, phase 18 and for TURB_PROF, LAM_PROF, and LAM_MEAN.

In order to compare our computational results with the experimental results in Fig. 8, we store contours at different sinusoidal phases during the simulation of the 75 cycles to produce the phase-averaged quantities. The phase-averaged vorticity results for phase 2, phase 10, and phase 18 are shown in Fig. 10. Note that the color map is chosen to match Fig. 8, i.e., it retains the nonlinearity and color discretization, which enables direct comparison with the experiments. The color map discretization, with only a few values, made the lower values of vorticity close to 10 Hz to be more pronounced. In the same way, experimental contours also presented some noise especially on the top half of the plane and for the heated condition, indicating the existence of non-zero values for the vorticity field. Finally, a small region of the head was visible, in the measurement window, in the top left corner of the images. This shows the vorticity produced by the thermal body in the region near the head.

The results in Fig. 10 show a reasonably good agreement with the experimental values in Fig. 8 for TURB_PROF, which is the approach that represented the vorticity more accurately. This is mainly attributed to the impact of the turbulent fluctuations on the jet development. LAM_MEAN and LAM_PROF failed to reproduce the evolution of the breath jet within the measurement window. These contours give an indicator of the jet breakup that can be appreciated for phases 10 and 18 and is only captured by TURB_PROF. The distance at which this occurs is shorter in the experiments, although the inlet model of TURB_PROF is the one that better captures the spatiotemporal evolution of velocity at the mouth. The assumptions imposed on the Reynolds stresses as well as the LES turbulence modeling can be responsible for producing these differences. For instance, Rodriguez^104^ showed the effect that DNS, LES, and Reynolds-average Navier-Stokes (RANS) turbulence models had on a turbulent jet. DNS, by resolving the entire eddy spectrum, showed a more chaotic behavior with an early jet oscillation. The LES results with different turbulent boundary conditions highlight the importance of representing the exhalation process. The modeling choices we outlined are a compromise between output efficiency, computational effort, and available data. It is worth noting that this approach was able to reproduce phases at the beginning, middle, and end of the exhalation process of a sinusoidal wave, which is particularly important as acceleration and deceleration of the mean velocity were present in the process. Further improvements to the state of the art of turbulence modeling will benefit the accuracy with which this problem can be solved at a large scale.

Some differences between experiments and simulations were noticed for TURB_PROF with the plume shifted slightly upwards. This suggests a stronger influence of the body plume on the exhalation for the different phases, but in particular, for phases 2 and 18. An important reason for this discrepancy may be the lack of a more accurate representation of the spatiotemporal Reynolds stresses at the mouth. However, there are other factors to take into account inherent to the experimental configuration. For instance, the influence of the shape of the body could cause local differences on the body plume affecting the interaction with the breath plume. The shape of the manikin in the experiment, with head and body slightly inclined forward, might produce a stronger effect on the breath plume. This affects all phases but is more relevant for early and final phases where the buoyancy is dominant.

**FIG. 11.**
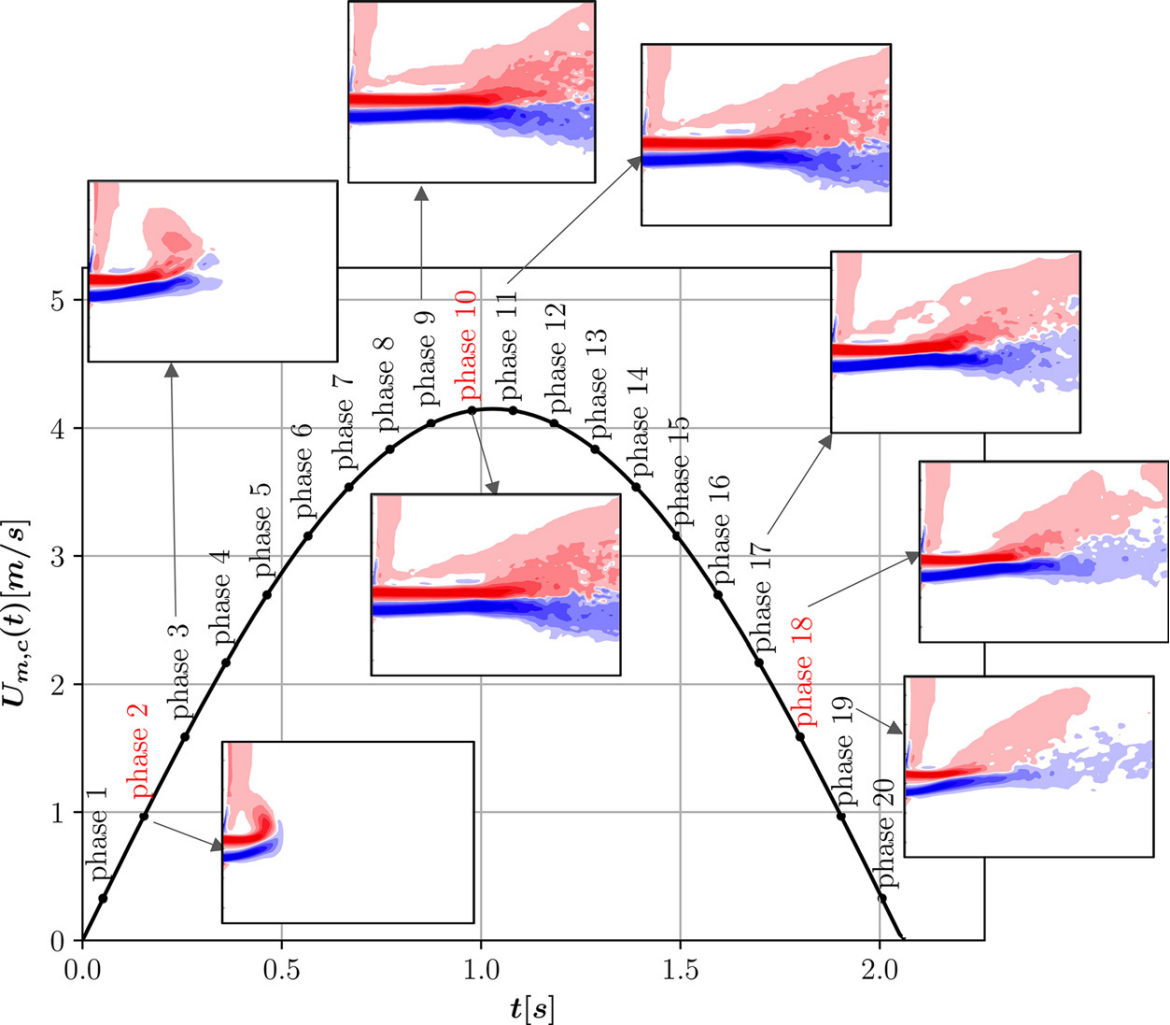
Phase-averaged vorticity contour evolution for different phases in TURB_PROF simulation. Phases in red represent the ones compared with experiments. Color maps are the same used in Fig. 10 for comparison.

In addition, errors on the sinusoidal fitting of the injected mean velocity is another source of uncertainty and has an impact on the comparison of specific phases. Figure 11 shows different snapshots of phase-averaged vorticity during the cycle for TURB_PROF, giving a better visualization of this evolution. Additional phases than the ones used for the validation with experiments are given in this figure. We can appreciate the variation of results around phases 2, 10, and 18. As described above, phases 2 and 18 are more affected by these uncertainties and the results at neighbor phases give a better baseline for the comparison.

Experimental and computational future work will help gain a better understanding of this process but will require detailed knowledge of the flow field of the upper tract section with measurements of Reynolds stresses at the mouth. In addition, experiments with a thermal manikin with detailed fully reported dimensions in detail will contribute to reproducing the body plume more accurately.

### C. Drop dynamics for vocalization

Section III B showed the capability of the model to represent the exhalation jet. We focus now on the investigation of drop dynamics for the vocalization virtual experiments. As Sec. III B, we include in this study the effect that the different modeling strategies of the inlet have on the dynamics of drops as represented with simulations LAM_MEAN_A, LAM_PROF_A, and TURB_PROF_A. A total of 410 drops were injected into the system for every simulation.

**FIG. 12.**
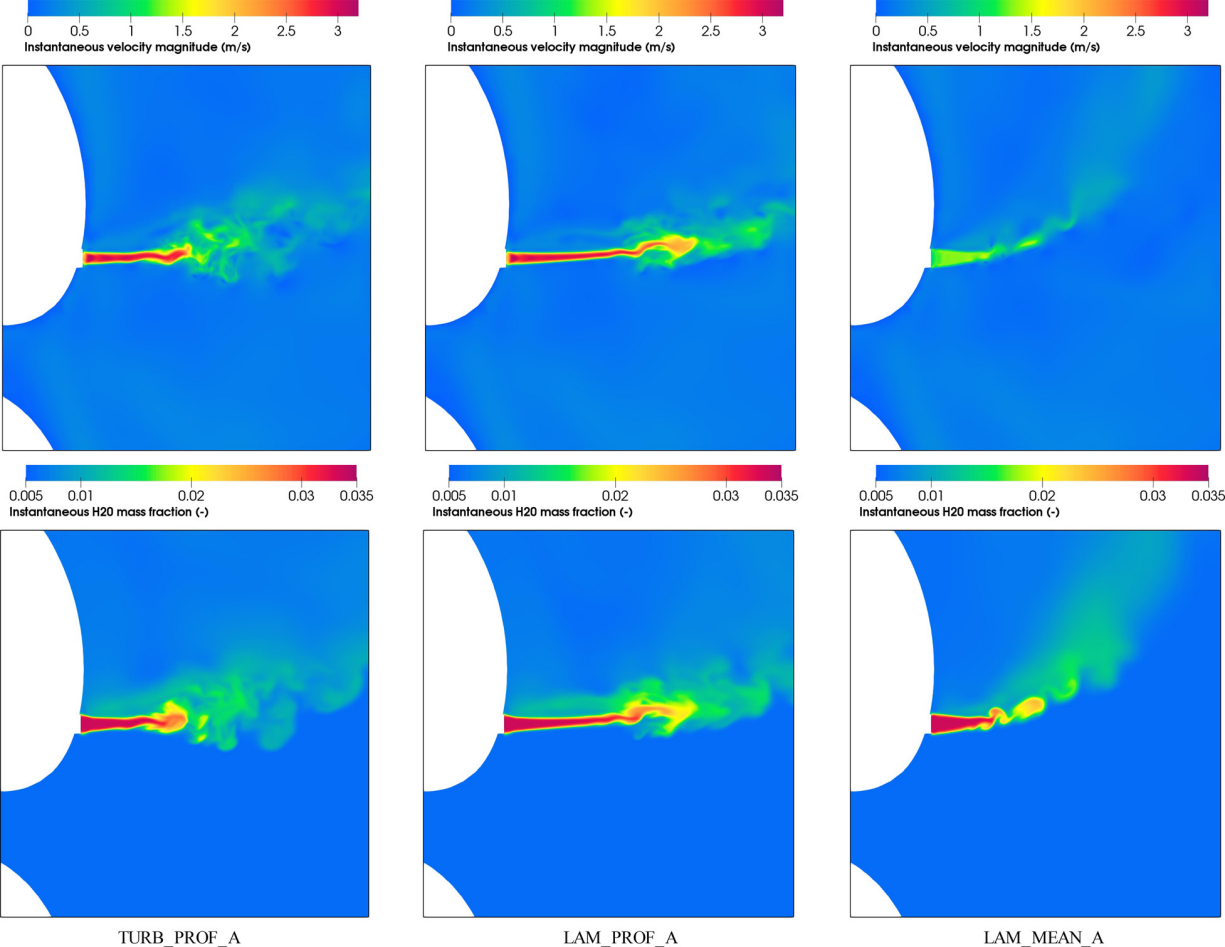
Instantaneous velocity and H_2_O contours for LAM_MEAN_A, LAM_PROF_A, and TURB_PROF_A at 207.5 s.

We analyze first, in Fig. 12, the contours for instantaneous velocity and H_2_O mass fraction variables for the vocalization of vowel /ɑ/and the different boundary conditions at a given time. The results show a more perturbed field near the mouth compared with the exhalation breathing in Fig. 9. This is in part because of the more intermittent flow produced during the vocalization exhalation. The comparison between the three different inlet boundary condition approaches shows significant differences in the flow field that anticipate an impact on the resulting drop dispersion. The plume generated in LAM_MEAN_A indicates that the drops will move toward the top of the domain at a shorter distance from the mouth compared with TURB_PROF_A and LAM_PROF_A. It is also expected that the spread of drops in TURB_PROF_A and LAM_PROF_A will be more pronounced because of the instabilities produced in the flow field, observed earlier for TURB_PROF_A. This effect will be also enhanced by the spatial in-homogeneity that results from the radial profile inlet and produces an increase in the lift force because of the local shear flow.

**FIG. 13.**
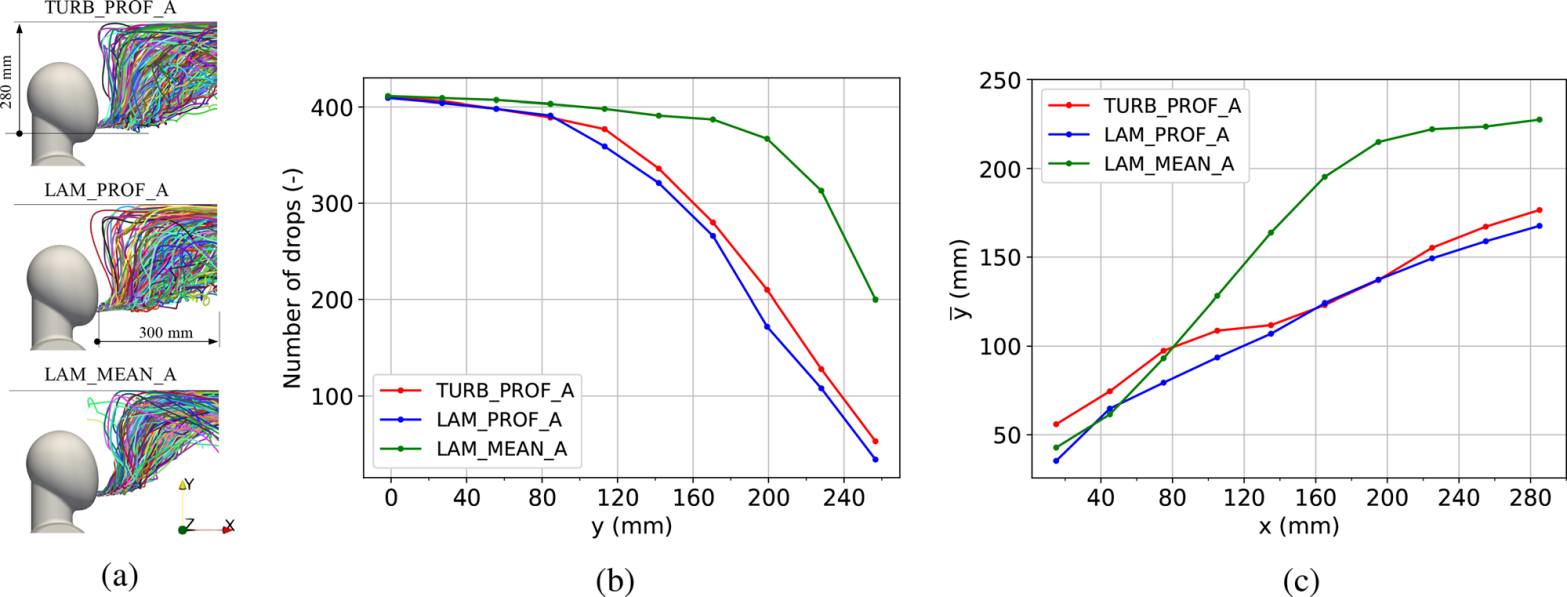
The effect of the inlet boundary condition on drop dispersion for the vocalization of vowel /ɑ/. (a) shows individual drop trajectories, (b) quantifies drop distribution in the vertical direction by counting unique drops that crossed equidistant windows positioned up to 280 mm above the mouth, and (c) quantifies the horizontal distribution through mean vertical drop position at a given streamwise coordinate up to 300 mm.

Figure 13 presents the drop dispersion produced during the vocalization of vowel /ɑ/ and the influence of the different inlet approaches evaluated. Figure 13(a) shows the paths followed by every drop during the duration of the simulation. The drop dispersion is also quantified by measuring the number of drops that crossed different vertical windows from the mouth to the top wall [Fig. 13(b)] and the mean vertical drop position along the exhalation streamwise direction for the first 320 mm [Fig. 13(c)]. Measurements at ten equidistant windows over *y* and *x* coordinates were performed.

**FIG. 14.**
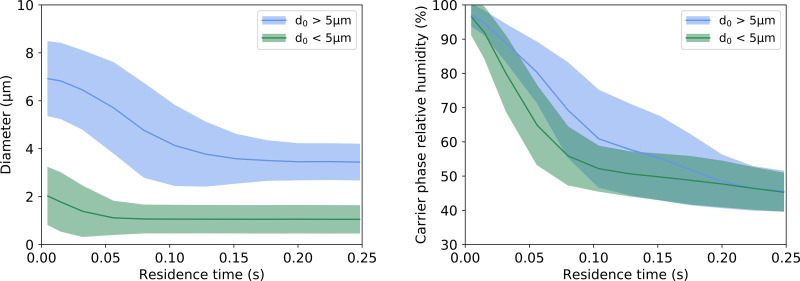
Diameter and carrier phase relative humidity as a function of drop residence time for two size groups for TURB_PROF_A. The shade area represents one standard deviation from the mean.

The results in Fig. 13 show that despite having a size distribution at the mouth up to 10 *μ*m, none of the drops deposited and all of them became part of the so-called aerosol. This is mainly because the evaporation process took place quickly from the release point while moving with the jet flow stream and reaching an equilibrium size below 5 *μ*m. The effect on the drop dynamics for laminar inlet cases was particularly pronounced for LAM_MEAN_A where drops followed a narrower plume compared to the other two approaches [see Fig. 13(a)]. LAM_PROF_A and TURB_PROF_A exhibited a wider distribution of drops. Figure 13(b) shows how the number of drops reaching a different vertical layer decreased with the vertical distance for this region. This image shows a similar behavior between LAM_PROF_A and TURB_PROF_A. In contrast, LAM_MEAN_A showed that almost all drops released from the mouth traveled to the region close to the top wall. Finally, Fig. 13(c) shows the mean vertical position of drops at a given distance along the exhalation direction. LAM_MEAN_A showed the biggest discrepancy compared to the other two. LAM_PROF_A and TURB_PROF_A differed mainly on the region close to the mouth. The presence of higher velocities, due to the velocity radial profile, caused the drops to travel longer distances within the jet before the buoyancy dominated the initial momentum. It is also important to note the effect that the turbulent inlet had on the drops' distribution. For instance, TURB_PROF_A shows a larger number of drops moving up and separating from the jet closer to the mouth, mainly because of jet breakup producing shorter jet cores during the exhalation process. This is in line with the observations made for the instantaneous velocity in Fig. 9 for the exhalation cycles experiment. This work shows the impact that the turbulent inlet had on the drops. A more precise definition of the spatiotemporal distribution of the Reynolds stress tensor at the mouth could result in a more pronounced difference between TURB_PROF_A and LAM_PROF_A, especially in the region below 150 mm described in Fig. 13(c). In any case, this work shows that the impact that the modeling of the mouth inlet boundary conditions might have when investigating virus transmission can be essential in certain scenarios. For instance, when evaluating social distancing or other mitigation measures in closed spaces, this might be especially relevant.

A deeper investigation was performed by analyzing the dynamics of the drops as a function of their residence time, or lifetime, in the system for TURB_PROF_A. Figure 14 shows diameter and carrier phase relative humidity evolution for particles with initial size, *d*_0_, bigger and smaller than 5 *μ*m. This evolution was obtained by averaging these variables over the drop residence time for each of the size groups.

The image shows how the diameter decreased over time due to evaporation and that it reached an equilibrium value within the domain. The mouth exhalation had a relative humidity of 99%, and therefore, evaporation was slightly delayed until later stages of drop trajectories. In the meantime, the drops interacted with the jet and turbulent eddies, and with the body plume. The evolution of the carrier phase relative humidity seen by the particles up to 0.15 s also indicated that drops bigger than 5 *μ*m remained under the influence of the exhaled jet for a longer time. As they evaporated and the buoyancy was predominant, both groups reached the humidity values of the environment.

## IV. CONCLUSIONS

This work showed a detailed and novel numerical investigation of drops released during a speech-like exhalation mode, i.e., a vocalization process. A breathing thermal manikin in a mockup was simulated using an LES coupled with Lagrangian tracking where every drop was represented as an individual entity. The simulations included transport of carrier species (air and water vapor), multicomponent particle tracking, carrier/particle vapor interaction, inflow synthetic eddy boundary conditions, heat transfer, and thermal radiation.

Prior to the release-of-drops experiment, vorticity contours were compared with experimental data for a set of exhalation breathing cycles. Results showed a good agreement in phase-averaged vorticity contours for different sinusoidal wave phases. We compared three approaches for modeling the velocity field boundary condition at the mouth. The results showed that only the approach representing a turbulent inlet (modeled with a synthetic eddy method) was able to properly capture the jet development and the turbulent field close to the mouth.

A complete model for reproducing the vocalization of a vowel /ɑ/ was applied to the conditions of a given female subject. Experimental data were used to define the exhaled velocity, size distribution, and particle rate for this case. The results showed the evolution of the drops as a result of their interaction with the jet as they evaporated. For this simulation, all the drops seeded became part of the aerosol and remained suspended within the air, reaching a maximum size below 5 *μ*m. Simulations for each of the three inlet boundary conditions evaluated also revealed how the effect of turbulence on the jet can lead to the presence of a higher number of drops closer to the head.

The presented results show the importance of an accurate modeling of the inlet condition at the mouth. Further experiments and simulations that provide accurate descriptions of the spatiotemporal turbulent characteristics at the mouth are recommended. For instance, high-fidelity simulations of the respiratory tract or coupled models for reproducing the phonation and airflow are needed for describing accurately and generalizing the seeding of particles for different speech-like exhalation modes. Dynamically accounting for mouth section variations, sound pressure signals, and airflow over time is needed to set up a more generic computational model.

This work demonstrated the capabilities of the model to reproduce vocalization scenarios. To achieve faster simulations on larger domains, our results could be used to calibrate less computationally intensive models e.g., RANS models or continuum models of the aerosol phase. Faster simulations can in turn benefit the uncertainty quantification studies that are required to account for the exhalation variability due to individual breathing patterns. Finally, this work will be extended to cover other scenarios where ventilation is a determining factor of virus transmission.

## Data Availability

The data that support the findings of this study are available from the corresponding author upon reasonable request.
